# mini-MED: study protocol for a randomized, multi-intervention, semi-controlled feeding trial of a Mediterranean-amplified vs. habitual Western dietary pattern for the evaluation of food-specific compounds and cardiometabolic health

**DOI:** 10.1186/s13063-024-07939-8

**Published:** 2024-02-02

**Authors:** Emily B. Hill, Minghua Tang, Julie M. Long, Jennifer F. Kemp, Jamie L. Westcott, Audrey E. Hendricks, Nichole A. Reisdorph, Wayne W. Campbell, Nancy F. Krebs, the mini-MED Trial Team, Claudia Schaefer, Gabrielle Glime, Katrina A. Doenges, Richard M. Reisdorph, Sakaiza Rasolofomanana Rajery, Kathryn Garvey, Robin Shandas, Sarah J. Borengasser, Daniel N. Frank

**Affiliations:** 1 Department of Pediatrics, Section of Nutrition, University of Colorado Anschutz Medical Campus, Aurora, CO 80045 USA; 2 Department of Biomedical Informatics, University of Colorado Anschutz Medical Campus, Aurora, CO 80045 USA; 3 Department of Mathematical and Statistical Sciences, University of Colorado Denver, Denver, CO 80204 USA; 4 Department of Pharmaceutical Sciences, University of Colorado Anschutz Medical Campus, Aurora, CO 80045 USA; 5 Department of Nutrition Science, Purdue University, West Lafayette, IN 47906 USA

**Keywords:** Nutrimetabolomics, Foodomics, Metabolomics, Biomarker, Diet assessment, Mediterranean diet, Microbiome, Randomized controlled trial

## Abstract

**Background:**

Diet is among the most influential lifestyle factors impacting chronic disease risk. Nutrimetabolomics, the application of metabolomics to nutrition research, allows for the detection of food-specific compounds (FSCs) that can be used to connect dietary patterns, such as a Mediterranean-style (MED) diet, to health. This validation study is based upon analyses from a controlled feeding MED intervention, where our team identified FSCs from eight foods that can be detected in biospecimens after consumption and may therefore serve as food intake biomarkers.

**Methods:**

Individuals with overweight/obesity who do not habitually consume a MED dietary pattern will complete a 16-week randomized, multi-intervention, semi-controlled feeding study of isocaloric dietary interventions: (1) MED-amplified dietary pattern, containing 500 kcal/day from eight MED target foods: avocado, basil, cherry, chickpea, oat, red bell pepper, walnut, and a protein source (alternating between salmon or unprocessed, lean beef), and (2) habitual/Western dietary pattern, containing 500 kcal/day from six non-MED target foods: cheesecake, chocolate frozen yogurt, refined grain bread, sour cream, white potato, and unprocessed, lean beef. After a 2-week washout, participants complete four, 4-week intervention periods, with biospecimen sampling and outcome assessments at baseline and at intervention weeks 4, 8, 12, and 16. The primary outcome is change in the relative abundance of FSCs from the eight MED target foods in participant biospecimens from baseline to the end of each intervention period. Secondary outcomes include mean change in cardiometabolic health indicators, inflammatory markers, and adipokines. Exploratory outcomes include change in diversity and community composition of the gut microbiota.

**Discussion:**

Our stepwise strategy, beginning with identification of FSCs in whole diets and biospecimens, followed by relating these to health indicators will lead to improved methodology for assessment of dietary patterns and a better understanding of the relationship between food and health. This study will serve as a first step toward validating candidate food intake biomarkers and allow for assessment of relationships with cardiometabolic health. The identification of food intake biomarkers is critical to future research and has implications spanning health promotion and disease prevention for many chronic conditions.

**Trial registration:**

Registered at ClinicalTrials.gov: NCT05500976; Date of registration: August 15, 2022.

## Administrative information

Note: the numbers in curly brackets in this protocol refer to SPIRIT checklist item numbers. The order of the items has been modified to group similar items (see http://www.equator-network.org/reporting-guidelines/spirit-2013-statement-defining-standard-protocol-items-for-clinical-trials/).

**Table:** 

Title {1}	mini-MED: study protocol for a randomized, multi-intervention, semi-controlled feeding trial of a Mediterranean-amplified vs habitual Western dietary pattern for the evaluation of food-specific compounds and cardiometabolic health
Trial registration {2a and 2b}.	This trial has been prospectively registered at ClinicalTrials.gov under the identifier: NCT05500976; Registered on August 15, 2022; Registered trial name: Metabolomics Initiative: Mediterranean-amplified vs Habitual Western Diet on Food Signatures, Health, and Microbiome (mini-MED); Local ethics committee registration approval number: COMIRB #21–4563
Protocol version {3}	This study protocol was based on version date August 24, 2023.
Funding {4}	This trial is supported by 5R01DK113957 (to Drs. Krebs, Campbell, and N. Reisdorph), the Institutional Training Program in Nutrition at the University of Colorado (5T32DK007658), and the Beef Checkoff of the National Cattleman’s Beef Association (NCBA). Data collection visits and outcome assessments at the CTRC and use of REDCap are supported by NIH/NCATS Colorado CTSA Grant Number UL1 TR002535. A research agreement between EnteroTrack and the study team at the University of Colorado Anschutz Medical Campus was signed, and EnteroTrack provided EnteroTracker® sampling devices free of charge.
Author details {5a}	^1^Department of Pediatrics, Section of Nutrition, University of Colorado Anschutz Medical Campus, Aurora, CO 80045, USA ^2^Department of Biomedical Informatics, University of Colorado Anschutz Medical Campus, Aurora, CO 80045, USA ^3^Department of Mathematical and Statistical Sciences, University of Colorado Denver, Denver, CO 80204, USA ^4^Department of Pharmaceutical Sciences, University of Colorado Anschutz Medical Campus, Aurora, CO 80045, USA ^5^Department of Nutrition Science, Purdue University, West Lafayette, IN 47906, USA ^*^Corresponding author
Name and contact information for the trial sponsor {5b}	Department of Health and Human Services, National Institutes of Health (NIH), National Institute of Diabetes and Digestive and Kidney Diseases (NIDDK), healthinfo@niddk.nih.gov, 1–800-860–8747
Role of sponsor {5c}	The sponsors played no role in study design, collection, management, analysis, interpretation of results, writing of the report, or the decision to submit the report for publication. Contents are the authors’ sole responsibility and do not necessarily represent official NIH views or views of the NCBA.

## Introduction

### Background and rationale {6a}

The sustained modification of diet provides perhaps the single most important opportunity to influence an individual’s health throughout their lifetime. Consistent findings demonstrate consumption of fruits, vegetables, legumes, nuts, whole grains, and seafood within healthy dietary patterns such as a Mediterranean-style (MED) dietary pattern has several health benefits and may contribute to reducing risk factors for cardiometabolic disease and increasing longevity [1, 2]. Great efforts have been made to test the effectiveness of isolated nutrients and phytochemicals from dietary patterns such as MED as well as specific foods consumed within complex background diets [3]. However, weak and often inconclusive findings connecting specific dietary intakes to health outcomes are frequently observed and could be due to the heterogeneity of study designs, lack of high quality randomized controlled trials in nutrition research, variations in dosage and timing of interventions, limitations of dietary assessment methodologies, or complexity of dietary patterns, among others [4–7]. It could also be that multifaceted interactions of foods and food metabolites, rather than individual nutrients or compounds, are required for health benefits. Hence, to improve our understanding of the relationship between diet and health, a step-wise strategy is warranted, beginning with identifying compounds that are unique to specific foods in biospecimens followed by determining associations between these signatures, dietary intakes, and health [8–10].

Our group has employed such a strategy to identify food-specific compounds (FSCs) that are detected in biospecimens, associate with intake of that food, and are associated with cardiometabolic health indicators [11–13]. Our findings suggest that several foods consumed within complex healthy dietary patterns such as the Dietary Approaches to Stop Hypertension (DASH) and MED diets have novel food-specific signatures [11–13]. To date, we have conducted metabolomics analyses of over 100 foods to identify compounds uniquely present in these foods. Using the established platform and methodology developed by our team [14, 15], we have identified thousands of distinct compounds within individual foods present in a MED dietary pattern [16], demonstrating our ability to use our approach to define FSCs and suggesting these FSCs may be further explored as biomarkers of food intake. The relationships between these biomarkers of food intake and health effects can then elucidated.

In addition to identifying FSCs from individual foods, we have shown that these compounds are present in biospecimens such as blood and urine from individuals who have consumed these foods as part of a fully controlled feeding intervention [11, 13]. These results suggest FSCs are discernable in biospecimens after consumption of complex meals within overall dietary patterns and thus may be useful as putative biomarkers of food intake [17]. We have also demonstrated the ability to link observed FSCs in plasma with clinical indicators, suggesting food compounds may be associated with a biologic effect (i.e., putative biomarkers of effect) [11]. For example, the MED intervention improved participant systolic and diastolic blood pressures, and plasma/serum total cholesterol, triglycerides, and LDL cholesterol [16]. Results from our analyses identified FSCs that are associated with improvement in one or more of these cardiometabolic health indicators. These promising results warrant follow-up to confirm findings in an independent cohort.

Our results support the use of unmetabolized FSCs in either plasma or urine as candidate biomarkers of food intake. However, evaluation of multiple biospecimen types (e.g., blood and urine) simultaneously may increase coverage by collecting complementary information about different aspects of food metabolism [18]. Thus, collection and analysis of multiple biospecimen types will provide more comprehensive compound lists to define metabolomic signatures for testing associations with health indicators. Our preliminary results need to be prospectively tested within an independent intervention, a necessary next step for evaluation of reproducibilty and potential validation as biomarkers of food intake. Notably, our previous nutrimetabolomics research has been conducted within fully controlled feeding trials, making it difficult to elucidate the effects of incremental shifts between an “unhealthy” habitual/Western dietary pattern toward “healthy” evidence-based dietary patterns such as DASH or MED. The current mini-MED clinical trial study design and extensive biospecimen sampling plan directly address these limitations.

We are conducting a 16-week randomized, multi-intervention, semi-controlled feeding study among individuals with overweight/obesity who do not habitually consume a MED dietary pattern to comprehensively evaluate the reproducibility of FSCs using our innovative nutrimetabolomics approach. The novel FSCs identified in our eight MED target foods will be evaluated in participant biospecimens to assess change after intervention and to validate associations with cardiometabolic health indicators in this prospective semi-controlled feeding trial. The overall scientific premise is that FSCs will be reproducibly found when individuals consume specific foods, will not be found when individuals do not consume those foods, and will be associated with beneficial indicators of cardiometabolic health. In addition, we will evaluate the reproducibility of FSCs from beef in the context of different backgrounds (MED vs habitual/Western foods). Based upon existing literature, exploratory analyses will also be conducted to identify shifts in microbiome structure/function in response to incremental dietary changes.

### Objectives {7}

The primary aim of the study is to validate results from metabolomics analyses of foods and biospecimens from a completed MED-style dietary intervention in a prospective feeding trial. Our primary hypothesis is that pre-defined FSCs in participant biospecimens will be responsive to dietary intakes and reproducible within individuals over time, with beef FSCs stable and reproducible within the context of different dietary backgrounds (i.e., the MED and Western dietary patterns). Our secondary aims are to evaluate impacts of incremental changes in diet on cardiometabolic health indicators and microbiome structure and function. Our secondary hypotheses are that a Mediterranean-amplified dietary pattern will lead to improvements in cardiometabolic health indicators and beneficial changes in gut microbiome diversity and community composition over time and compared to a habitual/Western-style dietary pattern.

### Trial design {8}

**Fig. 1:**
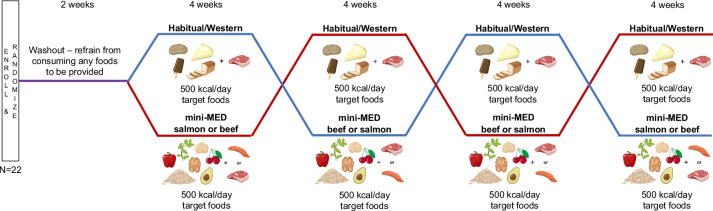
mini-MED clinical trial study design. After a 2-week washout, participants in the mini-MED clinical trial are randomized (1:1) to start one dietary intervention containing 500 kcal/day from target foods, with subsequent assignment to the other upon completion of the first. Each 4-week intervention is repeated once. The mini-MED arm contains target foods found within a Mediterranean-style (MED) dietary pattern and includes a second randomization to first receive either salmon or lean, unprocessed beef as a protein source, with consumption of the alternate protein source during the second mini-MED intervention period. The Western arm contains foods found within a habitual/Western-style (Western) dietary pattern with only lean, unprocessed beef as a protein source. All dietary intervention periods are 4 weeks in duration. Participants complete in-person assessments and data collection pre/post each intervention period (baseline and intervention weeks 4, 8, 12, and 16). Figure created with BioRender.com

This trial is a 16-week randomized, multi-intervention, semi-controlled feeding study of Mediterranean-amplified (mini-MED) and habitual/Western-style (Western) isocaloric dietary interventions containing 500 kcal/day from target foods. Participants are randomized (1:1) to start either the mini-MED or the Western dietary intervention, with subsequent assignment to the other upon completion of the first. Each 4-week intervention (mini-MED and Western) is repeated once. The Western intervention serves as both a washout of the mini-MED foods and a negative control for the postprandial response when the mini-MED foods are not consumed. Within the mini-MED arm, participants are further randomized to first receive salmon (mini-MED salmon) or lean, unprocessed beef (mini-MED beef) as a protein source, with consumption of the alternate protein source in their second mini-MED intervention period (Fig. 1). Outcome measures and biospecimen sampling take place at baseline and intervention weeks 4, 8, 12, and 16, and outcomes will be assessed using an exploratory approach.

## Methods: participants, interventions, and outcomes

### Study setting {9}

This study takes place at a single study site: the University of Colorado Anschutz Medical Campus in Aurora, CO, USA. The study protocol has been approved by the Colorado Multiple Institutional Review Board (COMIRB).

### Eligibility criteria {10}

To be eligible to participate in this research, volunteers must meet the following:

Inclusion criteria:

1. Body mass index between 25 and 37 kg/m^2^ and weight-stable;
2. Age 30–69 years;
3. Nonsmoker (including tobacco, marijuana, and other inhaled substances);
4. Self-reported consistent physical activity levels for 3 months prior to and throughout the study period;
5. Stable medication usage for 6 months prior to and throughout the study period;
6. Having at least one self-reported component of Metabolic Syndrome: i.e., elevated fasting glucose (≥ 100 mg/dL), elevated blood pressure (systolic ≥ 130 and/or diastolic ≥ 85 mmHg), elevated waist circumference (≥ 94 cm in males; ≥ 80 cm in females), elevated triglycerides (≥ 150 mg/dL), and/or reduced HDL cholesterol (< 40 mg/dL in males; < 50 mg/dL in females) OR on medication for the treatment of hyperglycemia, hypertension, or hypercholesterolemia [19];
7. Report of baseline consumption of a habitual dietary pattern not meeting criteria for a Mediterranean-style dietary pattern, defined as a score of ≤ 7 (on a zero to 14 scale of whole numbers) as assessed via the Mediterranean Diet Assessment Tool [20];
8. Follows an omnivorous dietary pattern and willing to consume all provided study foods;
9. Willingness to refrain from consumption of nutritional supplements, herbal supplements, botanical supplements, and pre- or probiotics aside from those prescribed by a physician for the duration of the study;
10. Willingness to come to the University of Colorado Clinical and Translational Research Center (CTRC) monthly for clinical assessments and biospecimen collection;
11. Willingness to participate in telephone interviews and data collections at the midpoint of the intervention periods;
12. No plans to relocate or for extended travel (greater than 1 week) within next 6 months; and
13. Capable and willing to provide written informed consent to this study and unspecified future research for data and donated biospecimens.

Exclusion criteria:

1. Current or recent participation in another research study (e.g., diet or weight loss intervention);
2. Use of medications contraindicating increased consumption of fruits/vegetables (e.g., warfarin);
3. Diagnosis of acute or chronic condition impacting appetite, food intake, and/or the metabolism and absorption of foods to be provided (e.g., Crohn’s disease, celiac disease, ulcerative colitis, short bowel syndrome, chronic diarrhea);
4. Impaired liver or kidney function;
5. Allergies to foods provided in either dietary intervention;
6. Vegetarian, vegan; and
7. For females: pregnant or lactating or planning to become pregnant during study period.

### Who will take informed consent? {26a}

Interested individuals can contact the study coordinator by phone or email or may follow a QR (quick-response) code/anonymous weblink to obtain more information and complete an eligibility screener via the secure online data collection tool, Research Electronic Data Capture (REDCap) [21, 22]. If they remain interested, individuals complete this screening assessment over the phone, by email, or via REDCap to determine preliminary eligibility based upon self-reported BMI and metabolic syndrome components and other inclusion/exclusion criteria as described above. A waiver of consent for screening allows for collection of contact information at this time. Individuals who are deemed conditionally eligible based upon this screening are contacted via their preferred communication method to schedule an in-person assessment.

After phone/email screening for basic eligibility, an eligibility/enrollment visit is scheduled and potential participants visit the University of Colorado Clinical and Translational Research Center (CTRC). A digital copy (PDF) of the consent form is emailed to participants for review prior to this visit. During the in-person CTRC visit, informed consent is obtained by the study coordinator using the e-Consent framework in REDCap [23]. The consent form is reviewed in a private setting, and potential participants are given the opportunity to ask questions prior to providing consent. Individuals must exhibit understanding by answering questions about the purpose of the study, participation requirements, risks, and benefits before providing informed consent. Those who refuse to provide informed consent at this time are discontinued from participation. Consented participants are registered with the Clinical Trials Office using the OnCore clinical trial management system (Advarra Research, Columbia, MD, USA).

### Additional consent provisions for collection and use of participant data and biological specimens {26b}

All enrolled participants provide blood, urine, and stool samples at the five in-person data collection visits conducted at baseline and intervention weeks 4, 8, 12, and 16. Data are stored and biospecimen samples are banked for unspecified future research.

### Interventions

#### Explanation for the choice of comparators {6b}

As this is a multi-intervention design, each participant serves as his or her own control to evaluate the primary outcome of detection of FSCs in biospecimens after consumption of each dietary intervention and reproducibility over time within an individual.

#### Intervention description {11a}

The dietary interventions are as follows: (1) Mediterranean-amplified (mini-MED) diet, containing 500 kcal/day from Mediterranean target foods: avocado, basil, cherry, chickpea, oat, red bell pepper, walnut, and salmon or lean, unprocessed beef as a protein source, and (2) habitual/Western (Western) diet, containing 500 kcal/day from non-Mediterranean target foods: cheesecake, chocolate frozen yogurt, refined grain bread, sour cream, white potato, and lean, unprocessed beef. Each intervention is 4 weeks, with a 2-week washout after enrollment but prior to initiation of the first dietary intervention (Fig. 1). Furthermore, each intervention is repeated to evaluate reproducibility within individuals over time.

Diets were designed to incorporate target foods identified from previous metabolomics analyses of 100 foods consumed within a completed MED controlled feeding intervention and representing distinct food groups emphasized (mini-MED) or limited (Western) in a MED dietary pattern. Our preliminary data suggest these foods contain FSCs that are detectable in participant biospecimens after consumption (Table 1). Furthermore, we selected foods that are widely accessible and available year-round and in combination will provide adequate energy to achieve 500 kcal/day. Total energy intake of approximately 3500 kcal/week (500 kcal/day) from these foods is provided. During the mini-MED intervention, participants are provided fresh conventionally-grown avocados, basil, red bell peppers, and beef (both ground 90% lean and beef tenderloin); frozen sweet cherries and Atlantic, farm-raised salmon; canned chickpeas; dried basil and old-fashioned oats; and shelled, unsalted walnuts. During the Western intervention, participants are provided New York style cheesecake and regular sour cream, fresh white potato and beef (both ground 90% lean and beef tenderloin), refined grain rolls, and chocolate frozen yogurt bars. Serving sizes and number of servings per week are determined using the US Dietary Guidelines 2020–2025 [24] and serving size information in the United States Department of Agriculture (USDA)-supported food composition database, FoodData Central (Table 2). Dietary interventions are isocaloric and are not designed to fully replace participants’ usual diets; participants are instructed not to change other aspects of their diet aside from accounting for the 500 kcal/day of foods provided. Participants are asked to refrain from consumption of target Western foods when on each mini-MED intervention and vice versa.

**Table 1:** Number of food-specific compounds detected in food and plasma for target foods in mini-MED study

Food group	Food	Compounds detected in food	Compounds detected in plasma
*mini-MED target foods*			
Fruit	Cherry	207	30
Fruit/vegetable	Avocado	1446	136
Vegetable	Red bell pepper	283	27
Herb	Basil	204	112
Nut	Walnut	313	23
Legume	Chickpea	41	17
Whole grain	Oat	185	10
Seafood^a^	Salmon	508	143
Lean meat^b^	Beef	276	76
*Western target foods*			
Red meat	Beef	276	76
Starchy vegetable	White potato	19	0
High-fat dairy	Sour cream	271	8
Sweets/dessert	Chocolate frozen yogurt	467	27
Sweets/dessert	Cheesecake^c^	N/A	N/A
Refined grain	Refined grain bread	56	5

Food-specific compounds identified in foods and plasma from the completed MED controlled feeding intervention provide mini-MED and Western target foods for evaluation of reproducibility within the mini-MED intervention

^a^Participants consume salmon as a protein source during the mini-MED salmon arm

^b^Participants consume lean, unprocessed beef as a protein source during the mini-MED beef arm

^c^Cheesecake is included in the target food list for the Western diet but was not included in the original MED controlled feeding intervention and, as a complex food, was not analyzed using metabolomics and thus food-specific compound data are not available (N/A)

**Table 2:** Serving size and energy density of target foods provided during the mini-MED study

Food	kcal per 100 g	Serving size	kcal per serving	Form(s)	Servings per week	kcal per week
*mini-MED target foods*						
Cherry	63	½ cup (75 g)	47	Frozen, unsweetened	7	329
Avocado	167	1/3 total (45.3 g)	76	Raw, California	6	456
Red bell pepper	26	½ cup, chopped (74.5 g)	20	Raw	7	140
Basil	23	2 Tbsp	5	Fresh	5	25
	233	1 tsp	2	Dried	Varies	10
Walnut	654	½ oz (14.18 g)	93	“English”	6	558
Chickpea	139	1/3 can (84.3 g)	117	Canned, drained solids	6	702
Oat	379	1/3 cup (27 g)	102	Oats, regular and quick, dry	6	612
Salmon^a^	208	4 oz (113 g)	236	Atlantic, farmed, raw	3	708
Beef^b^	176	4 oz (113 g)	199	Ground, 90% lean meat, 10% fat, raw	2	398
	246	4 oz (113 g)	278	Beef, tenderloin, steak, separable lean and fat, trimmed to 1/8″ fat, choice, raw	1	278
**Total energy intake per week (kcal/week)**						** ~ 3500**
*Western target foods*						
Beef	176	4 oz (113 g)	199	Ground, 90% lean meat, 10% fat, raw	2	398
	246	4 oz (113 g)	278	Beef, tenderloin, steak, separable lean and fat, trimmed to 1/8″ fat, choice, raw	1	278
White potato	77	1 medium potato	164	Potatoes, flesh and skin, raw	5	820
Sour cream	198	1 Tbsp	24	Cream, sour, cultured	7	168
Chocolate frozen yogurt	131	1 cup	228	Frozen yogurts, chocolate	4	912
Cheesecake	321	1/6 of 17 oz cake (80 g)	257	Cake, cheesecake, commercially prepared	2	514
Refined grain bread	321	1 roll	93	Rolls, dinner, sweet	5	465
**Total energy intake per week (kcal/week)**						** ~ 3500**

All serving size and energy density information for target foods and weekly number of servings are outlined for each dietary intervention period. Participants are asked to consume target foods provided during their prescribed arm (e.g., mini-MED or Western) for each dietary intervention period while refraining from consumption of target foods provided in the other arm

^a^Participants consume salmon as a protein source during the mini-MED salmon arm

^b^Participants consume lean, unprocessed beef as a protein source during the mini-MED beef arm

All target foods for both the mini-MED and Western interventions are purchased by study staff at a local grocery chain using a study-specific “pickup” account [25–27]. Briefly, generic foods matching descriptions in nutrient databases (e.g., FoodData Central, https://fdc.nal.usda.gov/) are identified and Universal Product Codes recorded for consistency in ordering across participants and from week to week. Study personnel place weekly grocery orders for participants for pickup at a predefined time at a grocery store at the participant’s preferred location in the Denver, CO, USA, metropolitan area or for delivery to their home. Participants pick up or have groceries delivered at the time and location specified on a weekly basis. Adequacy of the dietary intervention to meet energy and nutrient intakes is assured by stable weight status, which is assessed weekly using a provided body weight scale and monthly during in-person data collection visits.

#### Criteria for discontinuing or modifying allocated interventions {11b}

No modification of the dietary interventions is permitted, and participants are asked to consume all provided study foods in prescribed amounts for the duration of the study. Participants may voluntarily withdraw from this study at any time for any reason. Furthermore, the principal investigator (PI) may decide to suspend a participant’s participation for any reason, (e.g., if he/she/they refuses to participate in any required study procedures, no longer meets study inclusion criteria, or is lost to follow-up).

#### Strategies to improve adherence to interventions {11c}

We recognize the nature of a feeding intervention within a real-world environment may lead to difficulty with adherence. To improve adherence, the study team purchases and provides all foods for participant consumption and provides detailed descriptions of quantities/number of servings of each food to be consumed per week, including ideas for food pairings and basic recipes upon request. Participants receive guidance and basic education for implementing all phases of the dietary interventions and routine support from the study coordinator to assist in identifying facilitators and barriers to adherence with the interventions. The team also provides a weekly checklist to help participants track their consumption of study foods. Study staff are available by phone, text, and/or email for the duration of the study to assist with any issues that may arise in order to identify and respond to barriers to adherence. The continuity of study staff for the duration allows for rapport-building to increase support and provide accountability. Regular dietary assessment via 3-day food logs aids in monitoring adherence with each intervention. Importantly, we do not want nor expect participants to change any other aspects of their habitual diets aside from consumption or avoidance of the mini-MED and Western diet target foods, and therefore we expect this flexibility to improve overall adherence. To improve adherence with data collection, all participants are given a study calendar containing all planned study visits and sampling procedures for each time point upon enrollment. Written and oral instructions are provided to ensure comprehension, and email reminders are sent prior to each in-person visit. Mid-intervention check-in phone calls with the study coordinator are also completed during each dietary intervention period.

#### Relevant concomitant care permitted or prohibited during the trial {11d}

Concomitant medication is permitted if use was stable for at least 6 months prior to study enrollment and remains stable throughout the study. Participants are asked to refrain from consumption of nutritional supplements, herbal supplements, botanical supplements, and pre- or probiotics aside from those prescribed previously by a physician for the duration of the study.

#### Provisions for post-trial care {30}

The study team will arrange medical care for participants if they experience an injury that is caused by this research. However, they are not provided any compensation if they experience harm or injury from participating in the study.

### Outcomes {12}

The primary outcome for this clinical trial is change in the relative abundance of FSCs from beginning to the end of each dietary intervention period in participant plasma samples. Secondary outcomes include detection of FSCs in participant urine samples as well as mean changes in cardiometabolic health indicators (e.g., total cholesterol, LDL cholesterol, HDL cholesterol, triglycerides, insulin, glucose, systolic and diastolic blood pressure), inflammatory markers (e.g., C-reactive protein, interleukin-6, tumor necrosis factor-alpha, alpha-1-acid glycoprotein), and adipokines (e.g., leptin, adiponectin) over each dietary intervention. Exploratory outcomes include changes in diversity and community composition of the gut microbiota over each dietary intervention period.

#### Participant timeline {13}

See Table 3 for the schedule of enrollment, randomization, interventions, and assessments for all study participants.

**Table 3:**
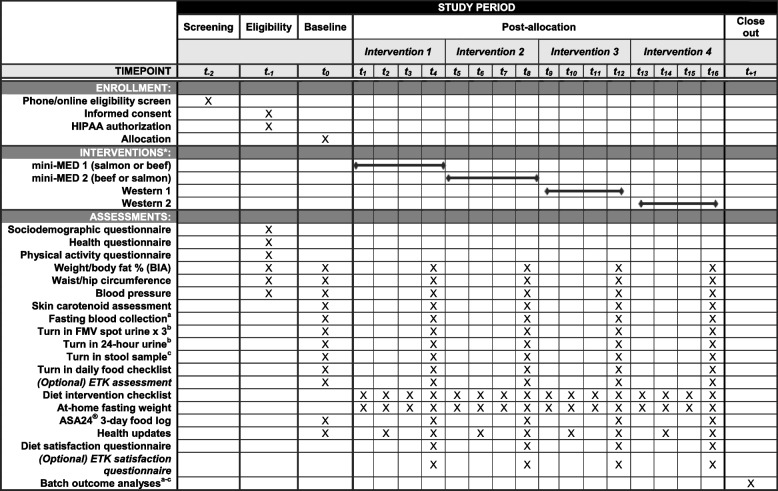
Schedule of enrollment, interventions, and assessments for mini-MED clinical trial participants

*Abbreviations*: *CTRC*, Clinical and Translational Science Center; *HIPAA*, Health Information Portability and Accountability Act; *BIA*, bioelectrical impedance; *FMV*, first morning void; *ETK*, EnteroTracker®; *ASA24®*, Automated Self-Administered 24-h Dietary Assessment Tool

^*^Order of intervention is randomized

^a^Blood will be processed for outcomes including: food-specific compounds identified through foodomics analyses; cardiometabolic health indicators (total cholesterol, LDL cholesterol, HDL cholesterol, triglycerides, insulin, glucose); inflammatory markers (e.g., C-reactive protein, interleukin-6, tumor necrosis factor-alpha, alpha-1-acid glycoprotein); and adipokines (e.g., leptin, adiponectin). Remaining samples will be stored for unspecified future analyses

^b^FMV spot and 24-h urine samples will be processed to assess putative biomarkers of dietary intake and food-specific compounds identified through foodomics analyses

^c^Stool samples will be assessed for diversity and community composition of the gut microbiota

#### Sample size {14}

Most of our planned analyses use each individual as their own control by looking at the change over each dietary intervention period. This reduces between subject variability compared to a cross-sectional analysis and increases the statistical power at a given sample size. To obtain a reasonable minimum expected effect size for the mean difference divided by the standard deviation of the difference (i.e., Cohen’s *d* effect size), we used our analysis of salmon FSCs [13]. The minimum salmon FSC effect size that passed an FDR adjustment was 0.45. Using a paired *t*-test after consuming the food, a sample size of *N* = 20 was needed to achieve 80% power for this effect size of 0.45 and a Bonferroni multiple testing adjustment for the number of compounds that increased in the preliminary MED data (*N*_compounds_ = 21; *p*-value_adjusted_ = 0.05/21 = 0.0024). We assume a 10% loss to follow-up based on our experience in similar dietary intervention trials and therefore enroll *n* = 22 eligible individuals to have *n* = 20 individuals complete the trial.

**Fig. 2:**
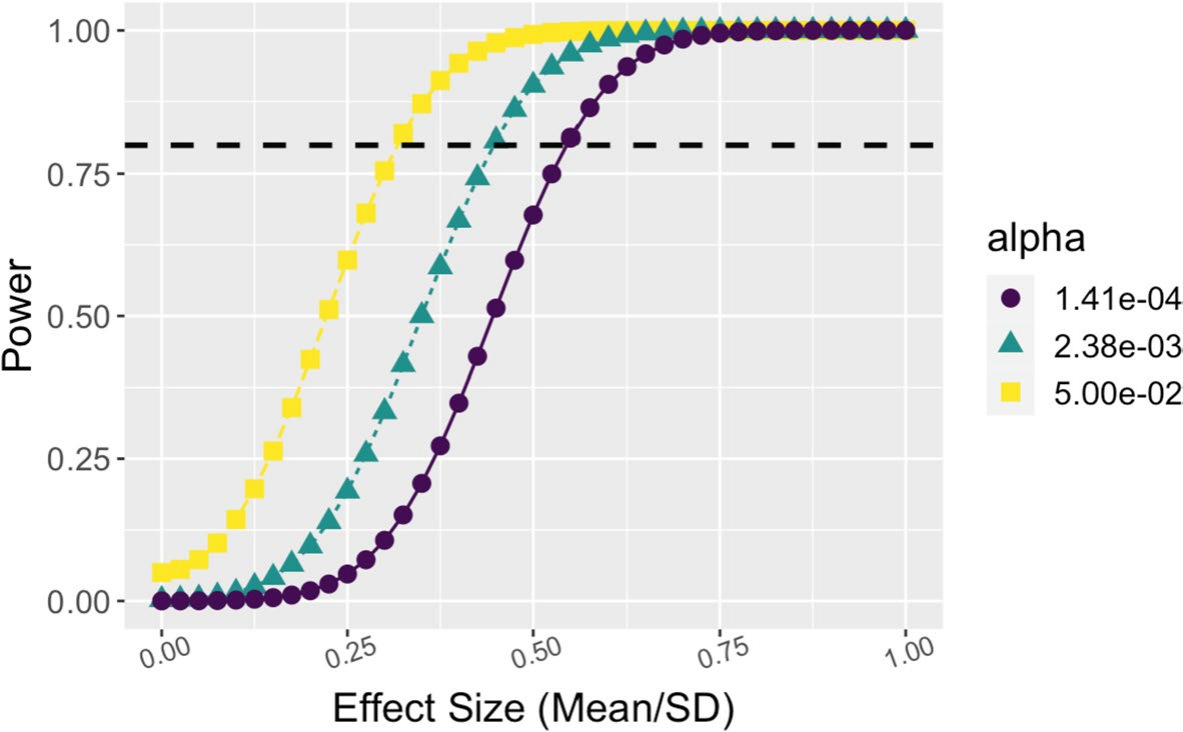
Power calculation for mini-MED clinical trial. Power (*y*-axis) for *N* = 20 for a paired *t*-test of the difference between post- and pre-consumption of a food. Power was evaluated over various effect sizes (*x*-axis, mean difference/SD of difference) for three significance thresholds: nominal threshold of 0.05 (yellow), Bonferroni corrected threshold for 21 compounds that increased after consumption in the initial study (*p* = 2.4e − 3), and Bonferroni corrected threshold for 355 compounds that were detected in MED foods and plasma (*p* = 1.4e − 4)

We also evaluated the power of *N* = 20 sample size further using the Bonferroni adjustment for the 21 compounds that increased after consumption of the preliminary Mediterranean diet foods (*p*_adj_ = 0.0024) and a stricter adjustment using all 355 compounds that were detected in the MED foods and plasma (*p*_adj_ = 0.05/355 = 0.00014). For both significance thresholds, we achieve power across a range of relevant effect sizes (Fig. 2) including nearly 100% power to detect an effect size of 0.75 (the median effect size for the salmon FSCs that passed the FDR threshold).

#### Recruitment {15}

Volunteers are being recruited from the Denver, CO metropolitan area. All participants are recruited using IRB-approved flyers posted on campus and at local community settings, the University of Colorado Anschutz Medical Campus Research Studies website, campus-wide email listservs, and ClinicalTrials.gov. Interested individuals may complete screening over the phone or by accessing a link to a screening questionnaire.

### Assignment of interventions: allocation

#### Sequence generation {16a}

All participants complete the mini-MED and Western dietary interventions twice each for four total intervention periods. Participants are randomized (1:1) to start either the mini-MED or Western dietary interventions, with subsequent assignment to the other upon completion of the first. A second randomization within the mini-MED arm is conducted to allocate participants to order of consumption of either salmon or lean, unprocessed beef as a protein source resulting in a total of four possible intervention orders (Fig. 1). Lean, unprocessed beef is consumed as the protein source in both Western intervention periods. A permuted block randomization scheme and stratification by sex has been generated and is utilized to ensure balance between intervention order and number of males and females. The allocation sequence was generated using computer-generated random number sequence in R using the *sample* command in *base* R package.

#### Concealment mechanism {16b}

Allocation of first intervention will take place after each consented individual is deemed eligible and enrolled. Participants are not notified of their intervention assignment until completion of baseline data collection.

#### Implementation {16c}

The randomization scheme is maintained and enacted by the study biostatistician, and participants are enrolled by the study coordinator after confirmation of eligibility.

### Assignment of interventions: blinding

#### Who will be blinded {17a}

Due to the nature of the dietary intervention, it is not possible to blind participants nor clinical trial staff responsible for carrying out the intervention, such as the study coordinator. Clinical trial staff are not blinded to the allocation sequence nor intervention assignment. However, outcome assessors responsible for data collection (e.g., assessment of vitals, anthropometrics, venipuncture) and those conducting clinical, metabolomics, and statistical analyses will be blinded, as all participants are provided, and all samples and data labeled with, a unique alphanumeric study identifier (ID) that is not associated with intervention order.

#### Procedure for unblinding if needed {17b}

Participants and clinical trial staff are not blinded; outcome assessors will remain blinded throughout the study.

### Data collection and management

#### Plans for assessment and collection of outcomes {18a}

As described above, during the eligibility/enrollment visit at the CTRC, participants complete informed consent and Health Insurance Portability and Accountability Act (HIPAA) authorization via the e-Consent framework in REDCap [23]. Those deemed eligible are provided materials by mail for at-home biospecimen sampling, including 24-h and spot urine collection containers, fecal collection kits, and items needed to transport the biospecimens to subsequent study visits for drop off. Future visits are also scheduled at this time. Participants complete data collection visits pre- and post- each dietary intervention period for a total of six in-person assessments (including eligibility/enrollment) over the duration of the 16-week intervention. The samples obtained and procedures conducted at intervention weeks 4, 8, and 12 will generate data that serve as both post-intervention data for the respective preceding intervention and pre-intervention data for the respective ensuing intervention. Mid-intervention phone calls with the study coordinator are conducted for each intervention period. Outcomes are assessed via standardized, self-report questionnaires that can be completed remotely or in-person at assessments at the CTRC as outlined below. Participants also complete several biospecimen collections throughout the study. Prior to each data collection visit, participants are asked to fast overnight (consume nothing except for water for a minimum of 12 h), to drink plenty of water the day before and morning of the visit, and to abstain from vigorous exercise and alcohol consumption for 48 h prior to the scheduled visit. Details regarding the sampling plan and data collection at each time point are outlined in Table 3.

##### Standardized, self-report questionnaires

Participants complete questionnaires remotely prior to or in-person at data collection visits.

1. *A sociodemographic questionnaire* is completed at the eligibility/enrollment visit. General demographic information, including age, sex, race, income, and education is collected as part of this assessment. Estimated time to complete this survey is under 5 min.
2. A *personal health assessment (PHA)* is completed at the eligibility/enrollment visit. Medical history is collected as part of this assessment. Estimated time to complete this survey is less than 10 min. Updates are collected in-person at the end of each 4-week intervention period to document changes in health data (e.g., change in medication, health status).
3. *Physical activity patterns* are assessed via the Global Physical Activity Questionnaire at the eligibility/enrollment visit and participants are asked to confirm that physical activity habits will remain stable throughout the duration of the intervention [28]. This is a validated measure of leisure-time physical activity and sedentary time, with resulting data providing estimates of activity in minutes/day and in metabolic equivalents. This questionnaire is a recommended physical activity assessment included in the NIH-supported Accumulating Data to Optimally Predict obesity Treatment (ADOPT) Core Measures project and takes approximately 5 min to complete [29].
4. *Dietary intake* is assessed via 3-day food records collected via the NIH-supported Automated Self-Administered 24-h (ASA24®) Dietary Assessment Tool, version (2022), developed by the National Cancer Institute, Bethesda, MD, USA. The ASA24® is a free, web-based tool that enables multiple, automatically coded, self-administered 24-h diet recalls and/or single or multi-day food records. Food records are employed in this study. Participants record three consecutive days of dietary intakes in real-time using this online tool at enrollment as well as pre-, mid-, and post- each intervention period. Time to complete these records varies based upon participant intakes. For pre- and post-intervention time points, participants align dietary intake assessment with biospecimen sampling, completing the final day of diet records the day prior to in-person assessment visits. Dietary data will be used to assess intake of target foods as well as overall dietary patterns such as adherence to the US Dietary Guidelines as measured by the Healthy Eating Index [30].
5. *Satisfaction with each dietary intervention period* is assessed after each intervention using both open-ended and closed-ended questions to gauge participant barriers to adherence, likes and dislikes about the foods included, and reported likelihood of adopting dietary behaviors after cessation of the intervention. This evaluation takes less than 5 min to complete.
6. *(Optional) evaluation of the EnteroTracker® upper gastrointestinal (GI) sampling device* is completed to assess participant experience with the device. This questionnaire probes for adverse events, side effects, and feelings of discomfort to assess feasibility and tolerability of the device. This evaluation takes less than 5 min to complete.

##### Anthropometrics and clinical assessments

Anthropometrics and clinical health indicators are assessed in-person at the CTRC pre- and post-each intervention period throughout the study as follows:

1. *Height/weight/body composition*: Standing height is assessed only at enrollment visits using a standard wall-mounted stadiometer by trained study personnel using standard Centers for Disease Control (CDC) protocols [31]. Care is taken to ensure individuals are standing in the proper position for standing height assessment with shoes removed, and height is recorded to the nearest 0.1 cm. Fasting weight is assessed in-person pre- and post- each intervention period using a calibrated TANITA Body Fat Analyzer TBF-105 digital scale equipped with bioelectrical impedance (BIA), which noninvasively assesses body composition (TANITA Corporation of America, Skokie, IL, USA). Participants are also provided a Bluetooth scale for home use to monitor fasting weight weekly for the duration of the study. Participants are asked to wear a provided standard procedure gown and remove shoes for all body weight assessments, and body weight is recorded to the nearest 0.1 kg. Height and body weight are used to assess BMI in kilograms divided by the square of height in meters.
2. *Waist and hip circumference*: Participant waist and hip circumference are completed pre- and post- each intervention period at in-person assessment visits at the CTRC using standard CDC protocols [31]. Participants are asked to stand straight with feet close together and hands resting at sides, wearing only light clothing. A plasticized tape measure is used to assess waist circumference between the costal margin and the iliac crest to the nearest 0.1 cm. Similarly, a plasticized tape measure is used to measure hip circumference at the maximum protrusion of the gluteus maximus. Three measurements for each circumference are taken and the averages recorded for analysis.
3. *Blood pressure*: Standard clinical methods are employed by trained study personnel to assess blood pressure using a manual sphygmomanometer on the right side of the body in a seated position after 5 min of rest. Three measurements are taken and results averaged for analysis.

##### Biospecimen sampling

At monthly in-person assessment visits at the CTRC, participants complete several biospecimen collections:

1. *Fasting blood draw*: Trained phlebotomists use standard sterile techniques to collect approximately 35 mL of venous blood into Vacutainer tubes with appropriate additives for specific tests (e.g., EDTA collection tubes for plasma analysis). Trace metal-free materials are used for all blood collection, processing, and storage. Samples are collected after 12-h fast at five time points throughout the intervention (pre- and post- each intervention). Samples will be used for analysis of specific outcome measures, including FSCs and cardiometabolic health indicators such as lipids, lipoproteins, inflammatory markers, and adipokines (described below). Blood collected for plasma carotenoid assessments is immediately covered with foil to protect from light and maintained on ice prior to processing.
2. *24-h urine sample*: Participants are asked to collect a 24-h urine sample the day prior to each in-person data collection visit (aligning with the final day of 3-day diet record collection). Briefly, participants are asked to start collecting urine after their first morning void on the day prior to in-person visits. All urine is collected using the materials provided (toilet hat, pre-labeled and pre-weighed 4 L high-density polyethylene (HDPE) jug) after this time and up until the first morning void the morning of the assessment visit, representing a full 24-h period. Urine is stored in a cool, dark place or refrigerated in the participants’ home refrigerators at approximately 4 °C between voids. Participants record start and end times and dates prior to placing in the provided cooler with ice pack for transport to the CTRC. Total urine volume is determined by weight, and samples are processed immediately to separate into aliquots, frozen on dry ice, and transferred to longer-term storage at − 80 °C for planned batch analyses at study completion.
3. *First morning void spot urine samples*: Participants collect 10–20 mL urine from their first morning void (FMV) for two days prior to and the morning of in-person data collection visits (including a small amount of urine not included within the 24-h urine sample) using a pre-labeled urine collection container. This aligns with days 2 and 3 and the day after 3-day diet record collection for a total of three FMV samples. Participants are asked to place their urine sample into their home freezer at approximately − 20 °C immediately after collection and to transport the sample in the provided cooler with ice pack to in-person visits at the CTRC. Samples are thawed on ice and processed immediately to separate into 2 mL aliquots and placed at − 20 °C prior to subsequent transport on dry ice to longer-term storage at − 80 °C for planned batch analyses at study completion.
4. *Fecal sample*: As close as possible prior to each in-person data collection visit, participants are asked to collect stool into the provided toilet hat and transfer to a pre-labeled fecal collection container using the provided collection tool/spatula and gloves (EasySampler® Stool Collection Kit, ALPCO Diagnostics, Salem, NH, USA) after training by research personnel. Participants record collection times and dates and place samples immediately in a home freezer at approximately − 20 °C upon collection. Participants are advised not to collect samples if taking certain medications (e.g., laxatives, antibiotics) or if experiencing diarrhea. Participants transport samples to the CTRC in the provided cooler with ice pack. Samples are thawed and processed immediately to separate into aliquots, frozen on dry ice, and stored at − 80 °C for planned batch analyses at study completion.
5. *Skin carotenoids*: Participant skin carotenoids are assessed noninvasively at each in-person assessment visit using the Veggie Meter® (Longevity Link Corporation, Salt Lake City, UT, USA), which is a research-grade instrument that detects and quantifies carotenoids in the skin through the use of pressure-mediated reflection spectroscopy [32]. Procedures follow the published recommendations for use of the Veggie Meter® in the research setting [32]. Briefly, participants are asked to wash hands with soap and water, and skin carotenoids are measured on the nondominant ring finger in triplicate. The average of three scan values, each lasting approximately 30 s, will be used for all analyses. Skin carotenoids have been validated against plasma carotenoids and used as a proxy for fruit and vegetable intake in several studies [33].
6. *(Optional) EnteroTracker® upper GI sampling device*: The EnteroTracker® is a novel, minimally invasive device developed at the University of Colorado for sampling the proximal gut that allows for the capture of samples from the upper GI tract and thus permits monitoring and study of the duodenal and jejunal microbiome, metabolome, and nutritional biomarkers. Externally, the 2-cm capsule device contains a slim, non-absorbent tethering string extending out of its end. Internally, it is a composed of a proximal, tethering string and a distal, collection string that are of different thicknesses and textures. This device is FDA class 1 510(k) exempt and cleared for commercial use for GI sampling. Participants have the option to complete the EnteroTracker® sampling pre- and post- each dietary intervention. Those choosing to participate are asked to refrain from use of mouthwash the morning of the visit where the EnteroTracker® device will be used. Briefly, when the intact capsule is swallowed, the tethering string is taped to the cheek. As the capsule passes through the upper GI tract, it deploys the collection string along the length of the esophagus, stomach, duodenum, and jejunum. The collection string then gently rubs on the epithelial surface obtaining a liquid biopsy from the mucosa. Dwell time for the string assessment is at least 1 h and no more than 4 h. Upon retrieval through the mouth, geographic localization of string samples is identified by (1) position along the length of the string (esophagus 0–45 cm, stomach 45-–65 cm, duodenum 65–90 cm, jejunum > 90 cm), (2) colorimetric changes on the collection string associated with changes in pH, and (3) presence of bile staining as an objective indicator of localization in the small intestine. Samples of the collection string corresponding to each segment are processed for planned microbiome assessments (e.g., 16S rRNA gene profiling, metagenomics). Previous studies have shown the EnteroTracker® is easily performed by a research assistant and well tolerated by patients, preferred over endoscopy, and thus highly suitable for repeated, unsedated, clinical and research sampling [34].

#### Plans to promote participant retention and complete follow-up {18b}

Efforts to promote participant adherence, as described above, are also expected to increase participant retention. In addition, study staff are available for the duration of the study to identify and respond to barriers to continued participation. The Western dietary pattern intervention period (4 weeks) is considered adequate for the participants to return to baseline in terms of the metabolic health status, as demonstrated by multiple cross-over studies [35]. Despite the 16-week study duration, our team has reported excellent adherence and retention in fully controlled feeding interventions (e.g., DASH and MED studies described above) of similar length, and therefore we are confident individuals will complete the study with minimal dropout or nonadherence [16, 36].

#### Data management {19}

All data, including consent documentation, forms for in-person assessments (e.g., vitals, blood pressure, height, weight), and questionnaires are collected by study staff or self-reported by participants using REDCap and identified by each participant’s unique alphanumeric study ID. REDCap is a secure, web-based application designed to support data capture for research studies and hosted at the University of Colorado, providing (1) an intuitive interface for validated data entry, (2) audit trails for tracking data manipulation and export procedures, (3) automated export procedures for seamless data downloads to common statistical packages, and (4) procedures for importing data from external sources [21, 22]. Exported data for analysis will include only non-protected health information. Tracking of sample collection information and freezer storage location for biospecimens are managed using the TrackVia automated workflow platform (TrackVia, Denver, CO, USA). Dietary intake data are collected via participant self-report through the ASA24®. Study staff specify a unique numeric identifier for each respondent and download system-generated ASA24® usernames and strong passwords that they provide to respondents so that they may access the application.

#### Confidentiality {27}

We will maximize efforts to ensure confidentiality through the use of unique alphanumeric study IDs. Participants are identified throughout the research database by their study ID. Only the research team has simultaneous knowledge of participant identities, ID numbers, and study-related data for all participants in the study. Records and forms are kept in locked file cabinets in a secured room. Any information stored virtually is housed on a secure server and is only accessible to study key personnel who have the password to gain entrance into the REDCap, ASA24®, or TrackVia databases. All computer systems are password-protected against intrusion, and all network-based electronic communications of confidential information are encrypted. Information sent for analysis will not contain any identifiers and will be transmitted in a secure manner. Only study team members who have completed their institutional or the NIH human participants training are able to work on this project.

#### Plans for collection, laboratory evaluation, and storage of biological specimens for genetic or molecular analysis in this trial/future use {33}

As described above, participants will collect urine and stool at home prior to each in-person assessment visit at baseline and intervention weeks 4, 8, 12, and 16 (pre/post each dietary intervention period). In-person assessment visits will consist of fasting blood draws and optional upper GI sampling via EnteroTracker®. All biospecimens (blood, urine, stool) will be processed and stored at – 80 °C for batch analyses of primary and secondary outcomes upon study completion as well as unspecified future analyses to learn more about heart disease, inflammation, and gut health or other health problems.

## Statistical methods

### Statistical methods for primary and secondary outcomes {20a}

Assessment of the primary outcome of change in FSCs will be completed using the intercept of linear mixed effects models (LMMs) with change in FSCs (post–pre) as the outcome and a random intercept to control for repeated measures. Analysis will be stratified by mini-MED and Western interventions since we expect FSCs to increase during the mini-MED arm and to decrease during the Western arm. FSCs that significantly increase during the mini-MED interventions and significantly decrease during the Western interventions will be considered validated. We will adjust for multiple testing using false discovery rate (FDR) for the number of FSCs [37]. We will complete stratified analysis by intervention period to assess reproducibility of FSCs in increase (mini-MED) and decrease (Western). The relationship between cardiometabolic health indicators and diet intervention will be assessed using LMMs similar to that described above. The relationship between cardiometabolic health indicators and FSCs will be assessed using LMMs over all intervention periods with an interaction term for FSCs and diet (i.e., mini-MED, Western) to capture differential effects by diet.

Data for exploratory outcomes related to the gut microbiota will be evaluated to assess (1) change over dietary intervention, (2) association with cardiometabolic health indicators, and (3) descriptive differences between upper GI and stool microbiota. Data will be presented as sequence count data. For both 16S rRNA gene amplicon and metagenomic datasets, taxa or genes that differ in abundance or prevalence between intervention groups and pre- to post-intervention will be identified by negative binomial mixed effects regression, with the total sequence count for each subject as a covariate [38]. Similarly, associations between gut microbiota variables and continuous cardiometabolic health indicator variables will be assessed using LMMs controlling for total sequence count. Permutation-based analysis of variance (PERMANOVA) tests using common beta-diversity indices (e.g., UniFrac, Bray–Curtis) will assess between-group differences in overall microbial community structures. Exploratory analyses of gut microbiota data also will be performed using unsupervised ordination methods (e.g., principal coordinates analysis). Supervised classification methods (e.g., random forests) will be used to build models of cardiometabolic health indicators, with the goal of identifying particular microbial taxa, or host factors, that best predict the cardiometabolic health indicators. To control for multiple testing for FSCs, cardiometabolic health indicators, and microbial taxa/genes, we will use an FDR correction [37].

### Interim analyses {21b}

No interim analyses are planned for this trial.

### Methods for additional analyses (e.g., subgroup analyses) {20b}

While not formally powered to examine subgroup differences, we will analyze change in FSCs after each dietary intervention by covariates such as age, sex, and baseline value of the outcome.

### Methods in analysis to handle protocol non-adherence and any statistical methods to handle missing data {20c}

Data from all randomized participants will be analyzed using intention-to-treat. Compounds with more than 90% missing over all plasma or urine samples will be removed. Remaining compound values will be log2 transformed, and data will be corrected for batch and order effects using residuals from linear regression with FSC abundance as the outcome with order, batch, and order by batch interactions as predictors after removing outliers and values below the detection limit (i.e., missing). Outliers will be defined as having residuals from the model that are more than the third quartile plus 1.5*interquartile range or less than the first quartile minus 1.5*interquartile range. The resulting linear model will be used to predict compound values for all observations (i.e., including outliers). After batch and order correction, QC values will be shifted by the minimum observed value for each compound, and compounds with a high coefficient of variation (CV ≥ 0.3) for QC values will be removed. Given the expectation of meaningful and real zero values (i.e., in baseline plasma samples) and arbitrary quantities of untargeted metabolomics, adjusted values will be ranked within each compound to enable parsimonious inclusion of zero values. Missing values will be given the lowest ranks. In case of multiple missing values or duplicate normalized values, an average of ranks will be assigned.

### Plans to give access to the full protocol, participant-level data, and statistical code {31c}

This paper provides the full study protocol. Metabolomics data will be deposited in the NIH Common Fund’s National Metabolomics Data Repository (NMDR) website, the Metabolomics Workbench, and accessible directly via its project number following publication. Analytic code will be publicly and freely available without restriction at GitHub. Other data will be made available upon request pending review by the study principal investigators.

## Oversight and monitoring

### Composition of the coordinating center and trial steering committee {5d}

This single site, randomized, multi-intervention, semi-controlled feeding trial does not have a coordinating center or trial steering committee. The trial is primarily supervised by the site PI (NFK) with additional oversight by postdoctoral fellow (first author, EBH) and mPIs (co-authors, NAR, WWC). The program coordinator and study professional research assistants (mini-MED Trial Team members/co-authors, JLW, CS, GG, JFK) provide day-to-day organizational support for the trial, including regulatory support, participant recruitment, consent, coordination of dietary intervention delivery and tracking, scheduling and conduct of outcome assessments, data entry, and processing and storage of biospecimens. Oversight meetings are held with the clinical trial team biweekly to discuss trial conduct and progress, including recruitment and retention. Full team meetings with mPIs and study co-investigators are held biweekly on the off weeks of the clinical trial meeting to discuss any issues that arise.

### Composition of the data monitoring committee, its role and reporting structure {21a}

The dietary interventions, biospecimen collections, and other outcome assessments pose minimal risk to participants. Thus, a data monitoring committee was not formed for this study, as no serious adverse effects are expected. The site PI (NFK) is a physician and provides frequent monitoring. In addition, study staff regularly monitor for adverse effects and/or undesirable symptoms, which can be reported using a case report form on REDCap.

### Adverse event reporting and harms {22}

Adverse event information is captured on a case-by-case basis in real time. All protocol violations and adverse events will be logged on a case report form in REDCap and reviewed and evaluated by the study PI, who is an MD, within 72 h. Serious adverse events will be evaluated by the PI within 24 h. Prompt reporting of excessive protocol violations, adverse events, or serious adverse events to COMIRB and the NIH will be conducted as needed.

### Frequency and plans for auditing trial conduct {23}

The trial is monitored continually by the study PI, postdoctoral fellow, and other study staff during biweekly clinical trial meetings as described above. An annual report is submitted to COMIRB for continuing review, and an annual progress report is submitted to the study sponsors (NIH and NCBA).

### Plans for communicating important protocol amendments to relevant parties (e.g., trial participants, ethical committees) {25}

Any protocol changes that impact study procedures or risk to human subjects (e.g., changes to eligibility criteria) will be submitted for approval by COMIRB and reflected in a revised consent form that will be reviewed and signed by all active participants. Protocol changes will also be amended in ClinicalTrials.gov and reported to NIH in annual progress reports.

### Dissemination plans {31a}

Study findings will be shared primarily via publication in peer-reviewed scientific journals and via national and international scientific conference oral and/or poster presentations. All journal articles will be submitted to PubMed Central (PMC) per the NIH Public Access Policy. Additional results will be disseminated via University of Colorado Anschutz Medical Campus social media channels and/or the lay press for the general population. All data will be available as described in the “Plans to give access to the full protocol, participant-level data, and statistical code {31c}” section. No data with personal identifiers will be published as part of this clinical trial.

## Discussion

This will be the first study to prospectively assess FSCs identified via metabolomics analysis of a completed MED controlled feeding intervention in an independent cohort. FSCs from target foods consumed within a complex background habitual dietary pattern will be assessed as putative biomarkers of intake to these foods as well as explored as potential biomarkers of effect by examining associations with cardiometabolic health indicators and microbiome community structure and function. This will achieve the important step of validation of FSCs for a large panel of foods within a complex dietary background which, to our knowledge, has not been performed. Such evaluation is critical for the rigorous development and testing of dietary biomarkers and will catalyze the advance in knowledge of the role of dietary patterns, specific foods, and individual compounds on the physiology of metabolic health outcomes. Strengths include a rigorously designed randomized, multi-intervention dietary intervention with provision of target foods of interest to encourage participant adherence. Frequent and intentional sampling of multiple biospecimen types (blood, urine, stool) aligning with dietary intake data and clinical assessments will allow for detailed assessment of the relationships between dietary intake, FSCs, and cardiometabolic health indicators.

Upon completion of this study, we will have tested a stepwise strategy to serve as a first step in validation of candidate biomarkers of food intake within dietary patterns, allowing for assessment of associations with cardiometabolic health indicators and thus laying the groundwork for future studies designed to assess uptake and depletion kinetics, dose–response, reproducibility, and interindividual variability. The resulting insights will enable us to inform definitive trials with improved clinical study design in the short term and implement precision nutrition interventions in the long term. The identification of biomarkers that link dietary intake to clinical status and biological functions is critical to future research in the following areas: vulnerable populations, including women of reproductive age and their offspring; comparisons of food- vs. supplement-based interventions; holistic treatment of diseases including cancer, HIV, and lung disease; diet-centric microbiome research; and food/agricultural science. We envision application of emerging technology to allow measurement of pre-identified FSCs in field-friendly platforms (e.g., blood spot cards). Such tools would allow investigations to monitor adherence and to identify responders and non-responders to beneficial food(s) and dietary patterns. Finally, with increasing recognition of the need for more sustainable dietary patterns, identification of FSCs to assess nutritional quality will be critical.

## Trial status

This study protocol is based on version date August 24, 2023. The study was open to accrual on August 5, 2022, and recruitment is ongoing. It is anticipated that recruitment will be completed in January 2024.

The approved informed consent documentation will be provided upon request.

## Data Availability

Only the study team will have direct access to the final analyzed data. As described above, metabolomics data will be deposited Metabolomics Workbench and accessible directly via its project number. Analytic code will be publicly and freely available without restriction at GitHub. Other data will be made available upon request pending review by the study principal investigators.

## Abbreviations

ADOPT: Accumulating Data to Optimally Predict obesity Treatment
ASA24®: Automated Self-Administered 24-h Dietary Assessment Tool
BIA: Bioelectrical impedance
BMI: Body mass index
CDC: Centers for Disease Control and Prevention
COMIRB: Colorado Multiple Institutional Review Board
CTRC: Clinical and Translational Research Center
DASH: Dietary Approaches to Stop Hypertension
FMV: First morning void
FSC: Food-specific compound
HDL: High-density lipoprotein
HIPAA: Health Insurance Portability and Accountability Act
LDL: Low-density lipoprotein
MD: Medical doctor
MED: Mediterranean
NCBA: National Cattleman’s Beef Association
NIH: National Institutes of Health
NMDR: National Metabolomics Data Repository
PERMANOVA: Permutation-based analysis of variance
PGP: Phosphatidylglycerol phosphate
PHA: Personal health assessment
PI: Principal investigator
PMC: PubMed Central
RD: Registered dietitian
REDCap: Research Electronic Data Capture
TG: Triglyceride
USDA: United States Department of Agriculture
